# Improving clinical trial design using interpretable machine learning based prediction of early trial termination

**DOI:** 10.1038/s41598-023-27416-7

**Published:** 2023-01-04

**Authors:** Ece Kavalci, Anthony Hartshorn

**Affiliations:** Lindus Health, London, UK

**Keywords:** Computational biology and bioinformatics, Data mining, Data processing, Data publication and archiving, Databases, Machine learning

## Abstract

This study proposes using a machine learning pipeline to optimise clinical trial design. The goal is to predict early termination probability of clinical trials using machine learning modelling, and to understand feature contributions driving early termination. This will inform further suggestions to the study protocol to reduce the risk of wasted resources. A dataset containing 420,268 clinical trial records and 24 fields was extracted from the ct.gov registry. In addition to study characteristics features, 12,864 eligibility criteria search features are used, generated using a public annotated eligibility criteria dataset, CHIA. Furthermore, disease categorization features are used allowing a study to belong more than one category specified by clinicaltrials.gov. Ensemble models including random forest and extreme gradient boosting classifiers were used to train and evaluate predictive performance. We achieved a Receiver Operator Characteristic Area under the Curve score of 0.80, and balanced accuracy of 0.70 on the test set using gradient boosting classification. We used Shapley Additive Explanations to interpret the termination predictions to flag feature contributions. The proposed pipeline will lead to an optimised clinical trial design and consequently help potentially life-saving treatments reach patients faster.

## Introduction

Clinical research includes interventional and observational studies that use volunteers to test if a new drug, treatment or medical device is efficient and safe^1^. Clinical trial design is a significant aspect of interventional studies to facilitate an optimised assessment of efficacy and safety of interventions^2^. ClinicalTrials.gov contains over 400,000 clinical study records from 220 countries. Studies are registered to the website by the sponsor or principal investigator of the clinical study. As of 2022, 14.5% of interventional trials recorded in clinicaltrials.gov registry terminated prematurely^3^.

Clinical trials that fail prematurely due to poor study design are a waste of resources and deprive society of data for evaluating potentially effective interventions^4^. The cost of failed trials is larger in later phase trials, such as Phase 2 and Phase 3 trials, due to their larger sample sizes and investment in prior trials^5^. Hence, an optimised trial design process is important to ensure a successful clinical study, well used resources and ethical use of participants^4^.

The interest in AI (Artificial Intelligence)/ML (Machine Learning) in healthcare has been increasing but mainly focusing on healthcare delivery rather than clinical research^6^. The studies that focuses on clinical research are mostly focused on predicting trial outcomes rather than on the design of clinical trials^7,8^. By unlocking the potential of past data using machine learning, it is possible to reduce the time and cost spent on the trial design process.

## Related works

Several studies in the literature focused on predicting clinical trial success using machine learning algorithms based on structured and unstructured features collected from clinical studies. These studies focus on predicting early termination of clinical studies using trial characteristic data combined with unstructured data. Follett et al. combined structured and unstructured data to predict clinical trial termination^9^. The study used text mining to generate vector features from description fields of the studies in addition to basic study characteristics features. Selecting completed and terminated trials, a dataset of 130,000 studies was created from information that was available prior to the start of the study. They then used machine learning techniques to predict outcomes.

Research conducted by Elkin et al. used machine learning to predict clinical trial termination from data extracted from clinicalTrials.gov^10^. The study generated a dataset of 68,999 studies. 640 features were generated using document embedding, keyword features and trial characteristics. The purpose of their study was to understand clinical trial termination at a deeper level and achieve satisfactory prediction results**.**

## Contribution

**Figure 1:**
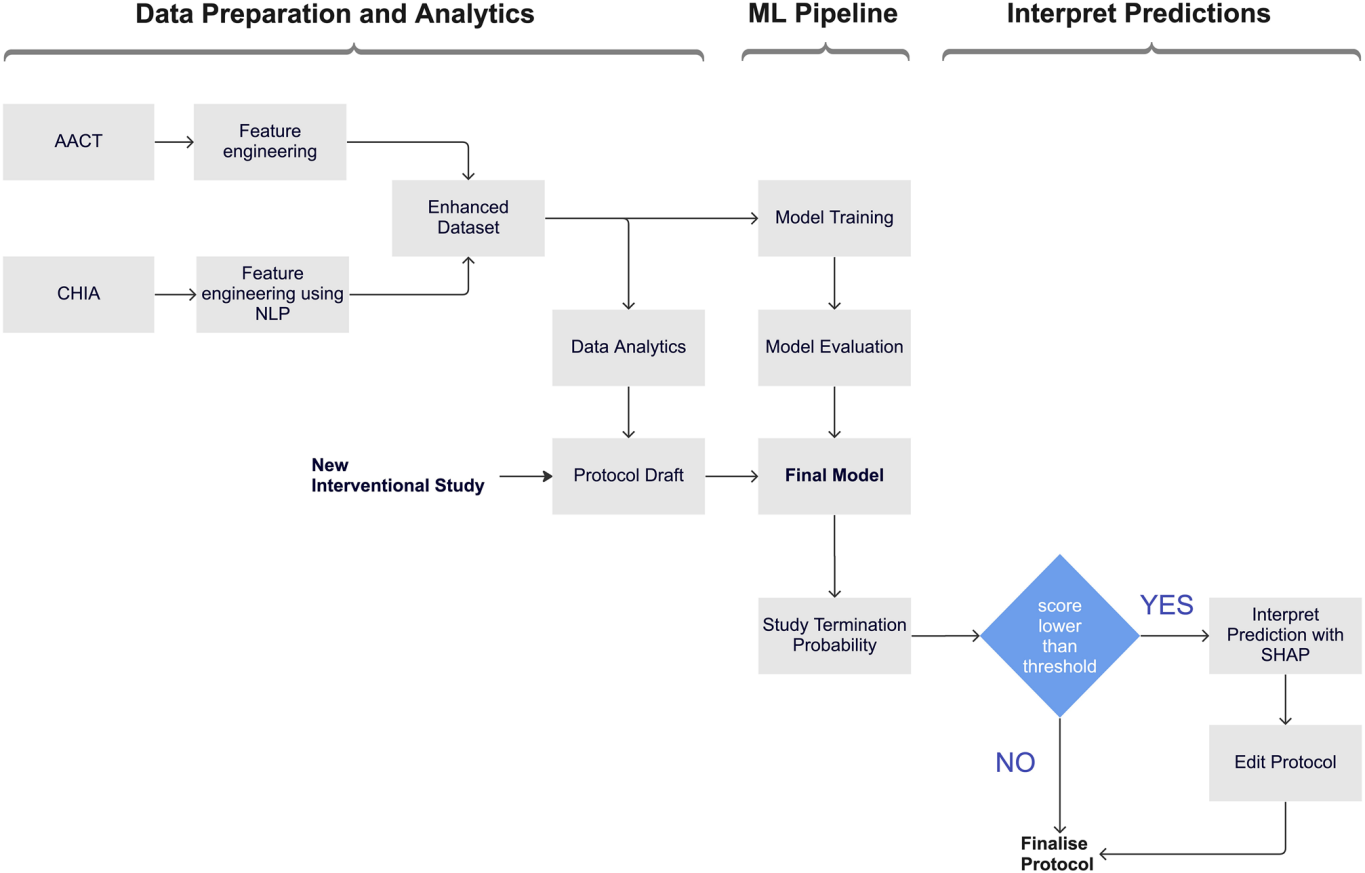
The proposed clinical trial design optimisation pipeline. This pipeline involves 3 stages: data preparation and analytics; machine learning (ML) pipeline and interpret predictions. This diagram summarises the generation process of the final ML model and the proposed usage of it in a real-world clinical trial design process.

We propose a machine learning based pipeline to validate and improve clinical study design as illustrated in Fig. 1. The main goal of this research is to investigate how big data analytics and machine learning based tools trained on big data can improve clinical trial design process.

Our research has notable contributions to the existing research in this field. Firstly, we generated an enhanced clinical trial dataset by transforming and integrating two public datasets. In addition to using only study characteristics features, we propose using a set of new features regarding eligibility criteria and disease categories. We generated eligibility criteria category/entity pair search features based on CHIA dataset^11^. We then added disease category features based on the clinicaltrials.gov categorization in which a study could belong to more than one category^3^. This enhanced dataset is to be published online to benefit further research.

We used this enhanced dataset to train machine learning models to predict early termination for any new interventional trial protocol. The last stage, “Interpret Predictions”, in the pipeline refers to the interpretation of the local prediction for the new study input in the pipeline using SHAP (SHapley Additive exPlanations). If the trial termination probability of the study input in the pipeline is lower than a defined threshold, prediction will be interpreted by providing feature contributions. This provides the clinicians with a set of flagged features to make further edits to the protocol. We provide individual feature contributions for every new clinical study protocol inputted to our pipeline whereas existing research analyses feature contributions for the overall machine learning model. This is an iterative process until the point that the optimised protocol is validated by the model and study clinicians.

## Methods

### Data source

The database for aggregate analysis of ClinicalTrials.gov (AACT) is a publicly available relational database enhanced by The Clinical Trials Transformation Initiative (CTTI) that contains both protocol and result elements for all studies recorded in ClinicalTrials.gov^12^. On 6 July 2022, the AACT database had 420,268 clinical studies registered from 1999 to July 2022, and this version is extracted in comma-separated values (CSV) format from the database for this research.

### Data preparation

Data preparation and analysis is a significant part of our proposed pipeline. There are three study types included in the raw dataset which are “Interventional”, “Observational” and “Patient Registry”. Only “Interventional” studies, the biggest proportion of the three study types, were filtered from the raw data.

Clinical trial success can have two different definitions; the success of the intervention or successful completion of the trial (whether the intervention achieved its objective/s). For the scope of this research, we use the latter definition. There are 14 study status types recorded for this dataset, including recruiting, completed, withdrawn and unknown statuses. The proposed pipeline aims to predict the probability of a study protocol leading to termination and, if so, interpreting this prediction to flag contributed features. Hence, studies that are completed, terminated or withdrawn were extracted from the original dataset. Our supervised machine learning algorithms will learn and output a simplification of these three statuses as a binary output of success or failure.

**Figure 2:**
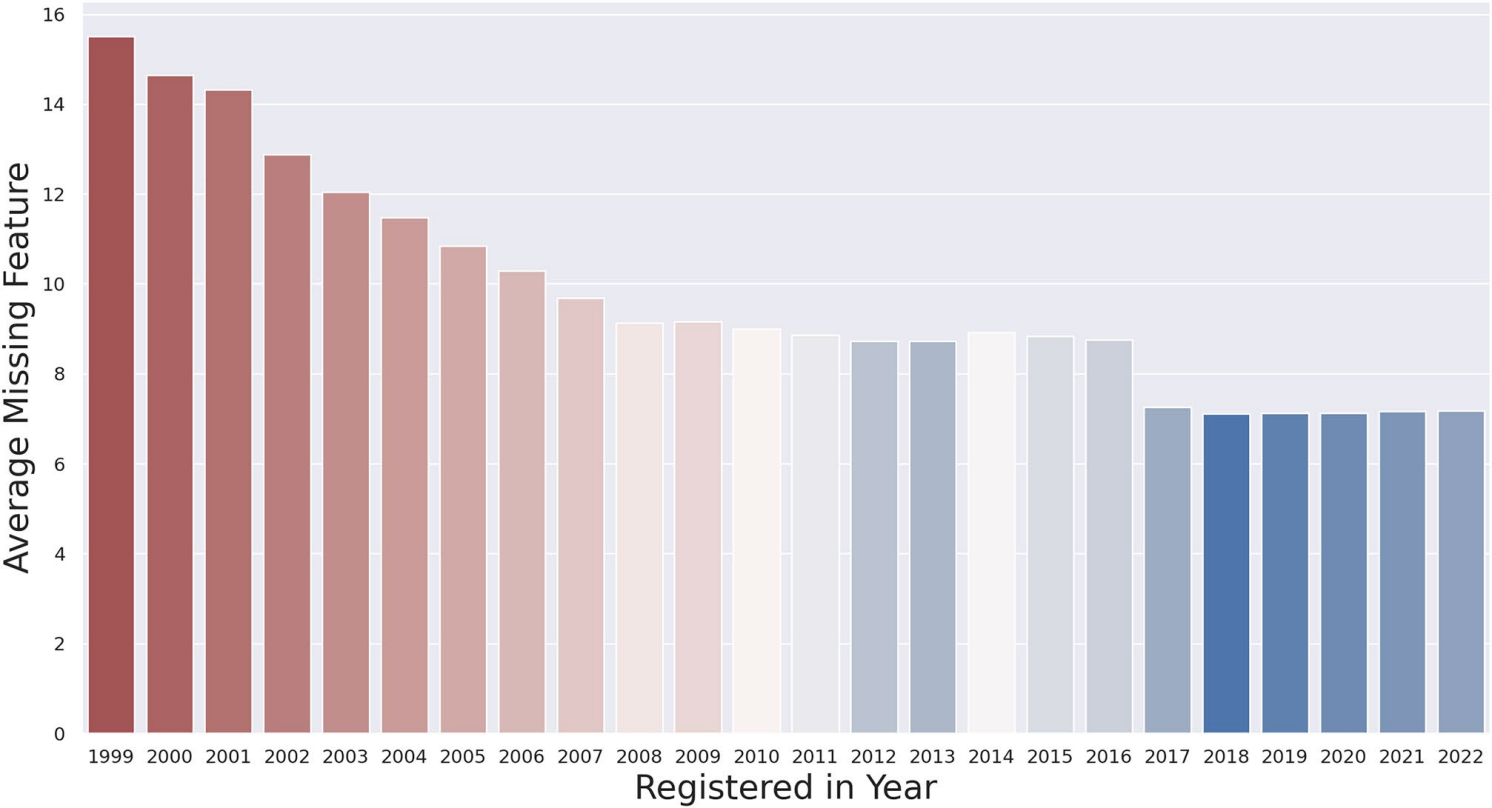
Average missing value rates (proportion of sum of missing values over total number of studies) per year from 1999 to 2022 considering the 24 study characteristics features used in this study.

Missing or erroneous data is common in real world big data sources. The objective of clinical study registries is to provide complete, accurate and timely recorded trial data. Although the emphasis on registering clinical studies and providing quality data increases over time (since 1999), there are still a high number of studies that have a significant number of missing data points or substantial errors^13^. Figure 2 illustrates a bar plot which shows the average missing value ratios starting from 1999, when the first study was registered to this registry, until 2022, considering the 24 study design features investigated in this initial analysis. Average missing values are calculated as the proportion of sum of missing values over the total number of studies recorded that year. There is a significant decrease in the average missing value rates over the years, especially from 1999 to 2008. After 2011, the average missing value rate is stable below 10. Hence, the studies registered before 2011 were removed from the dataset, leaving 112,647 studies.

### Studies by phases

Table 1 gives a summary of the number of studies and their recorded phases in the dataset. For some studies more than one phase can be recorded. In such cases, both phases considered correct during the generation of phase specific subsets. For example, if a study phase is recorded as “Phase2/Phase3”, it will be included in both Phase 2 and Phase 3 subsets.

**Table 1:** Study phases and number of studies recorded under each phase on clinicaltrials.gov.

Phase	Number of studies
Early phase 1	1549
Phase 1	23,914
Phase 1/phase 2	5350
Phase 2	25,599
Phase 2/phase 3	2910
Phase 3	19,699
Phase 4	16,463

### Study characteristics features

Study characteristics features includes logistic, administrative and design features of clinical trials. This section discusses some of the important features selected for the final feature set in more detail. The full list of numerical and categorical features is included in the supplementary materials.

Out of 190,678 studies, 19,252 did not record the number of sites. Although the recent growth in decentralised trials emphasises that sites are not always needed, most of the historical studies do not belong to this category of trials^14^. The number of clinical sites have an impact on trial enrolment and patient demographics, as the clinical study is limited by the participants who live near the defined sites and can attend study visits. Therefore, we used the number of sites in the final features set.

Defining the primary and secondary outcomes is an essential part of any interventional clinical trial^15^. The primary outcome measures directly form part of the study hypothesis. The number of primary and secondary outcomes to measure are included in our final feature set as two separate features.

A set of features specific to interventional studies, such as randomisation, intervention model, intervention type, masking and FDA regulation as a binary feature, are added to the final dataset.

### Disease category features

**Figure 3:**
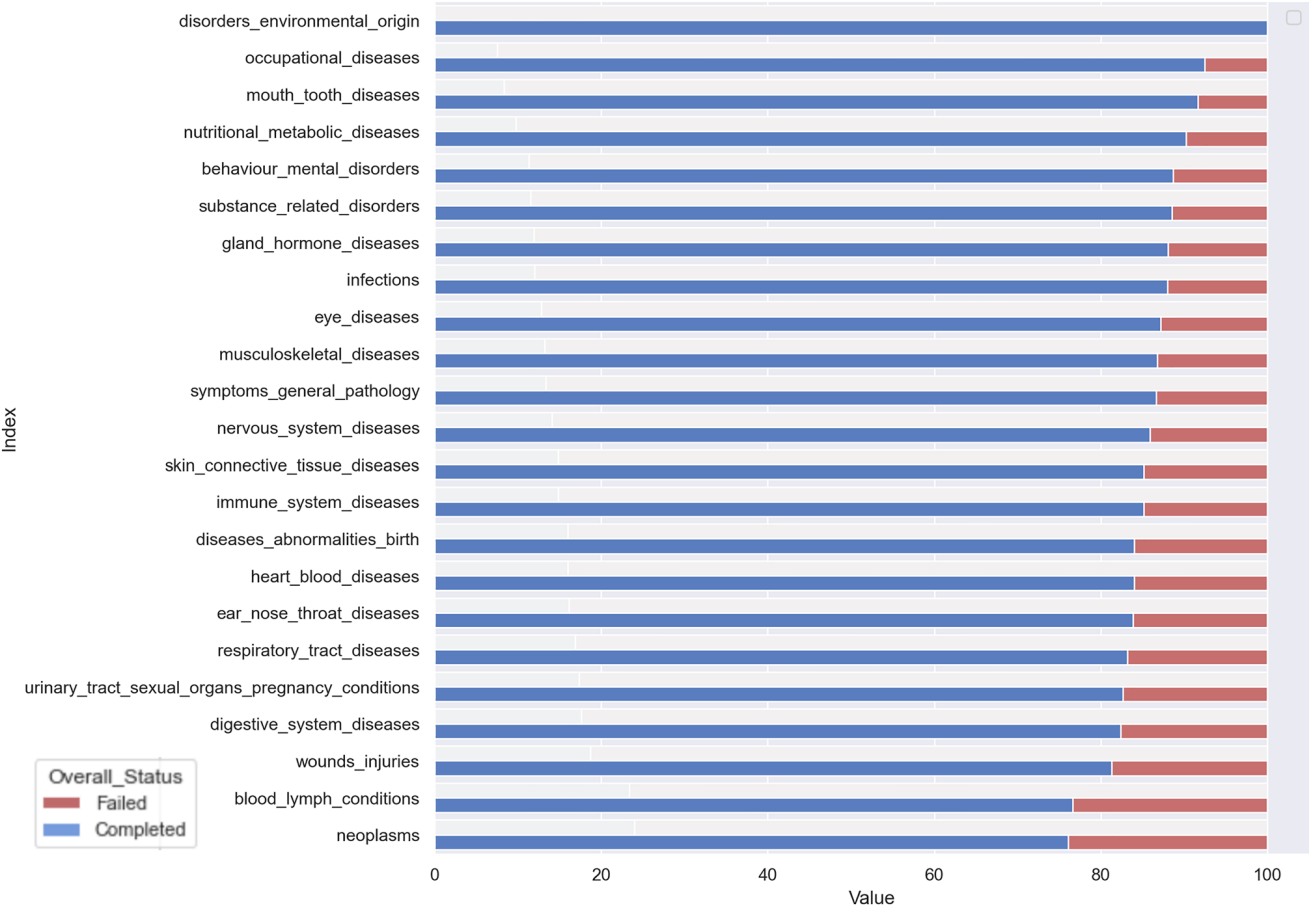
Percentage of completed to terminated studies for each disease category recorded on clinicaltrials.com.

Figure 3 illustrates proportion of completed to failed studies by disease category. Recorded conditions and mesh terms are combined to search for the diseases recorded under each disease category. This categorization of specific conditions is the same as that used in the clinicalTrials.gov database. As illustrated in Fig. 3, studies under neoplasms and blood lymph conditions categories are the most likely to fail, whereas studies under occupational diseases and disorders of environmental origin are the least likely to fail. The implementation of this categorization allows one study to be recorded under multiple disease categories.

### Eligibility criteria statistical and search features

Eligibility criteria is a free-text column in the raw dataset which includes inclusion and exclusion criteria specified in the study design. Eligibility criteria are implemented to control who can participate in clinical studies. Acceptance of healthy volunteers, and acceptance of patients by gender and age are among the features added, followed by number of inclusion and exclusion criteria, as well as total and average number of words for eligibility criteria per study. 54,758 studies that accepted healthy volunteers had a 7% failure rate, whereas 134,842 studies that did not accept healthy volunteers had a 17% failure rate. The importance of inclusive eligibility criteria has been emphasised increasingly over the years, as exclusion of particular subgroups makes it harder for studies to recruit patients and deliver inclusive outcomes^16^.

In addition to basic descriptive features generated from the eligibility criteria, our research introduces a set of more complex eligibility criteria search features generated using the public CHIA dataset by Kury et al.^11^. It is a large, annotated corpus of patient eligibility criteria extracted from 1,000 Phase IV studies registered in ClinicalTrials.gov. Annotating and generating search terms from the free-text eligibility criteria column in the original would result in a hugely manual and slow process with a massive output of search terms. Hence, we propose a more efficient way of generating search terms. The CHIA dataset contains 12,864 inclusion and exclusion criteria annotated with their entity category and value. We use the following category types in CHIA to generate our search terms: "Condition", "Procedure", "Person", "Temporal", "Drug", "Observation", "Mood", "Visit".

Category and entity pairs are generated for inclusion and exclusion criteria separately. 12,864 entity category and value pairs are generated as search features. The eligibility free text field in our dataset is separated into two fields, as inclusion and exclusion, and the generated search pairs are used to search the inclusion and exclusion fields from our original dataset. For computational efficiency reasons, we restricted search terms to those with 5 words or less and then search these using a 5-g language model in the original dataset. This process generated a sparse binary dataset of 12,864 features which concatenated to our original features.

### Data labelling

Overall status is recorded for every clinical trial in the AACT database. If no participants were enrolled in the trial, the status of that trial is ‘Withdrawn’, and if a trial was stopped prematurely, the status of that trial is ‘Terminated’. Out of 28,098 terminated or withdrawn studies that reported a reason for stopping the study, 9,260 studies prematurely stopped due to reasons related to participant recruitment and enrolment. Trials that are successfully completed have the status ‘Completed’. The classifying factor between studies for supervised machine learning model training is their overall status as being in either the success class or the failure class. Terminated and withdrawn studies are labelled as ‘failure’ and completed studies are labelled as ‘success’. “class 0” and “failure class”, “class 1” and “success class” will be used interchangeably.

### Numerical and categorical feature encoding

The final feature set is a mixture of numerical and categorical columns, which requires different methods of encoding. Large public datasets come with a lot of missing and erroneous data. Particularly for numerical features, handling of the missing values could have a big impact on predictive model performances. Multiple Imputation by Chained Equations (MICE) algorithm was selected to handle numerical missing data, as it is a robust and informative method^17^. Missing cells are imputed through an iterative sequence of predictive models where, in each iteration, one of the features is imputed using other features of the dataset. This algorithm runs until it converges, and all missing numerical feature values are imputed in this process.

One hot encoding is an effective method to encode categorical features. This method generates new binary features for each sub-category of a categorical feature. The method handles missing categorical values by encoding them as zeros.

#### Train/test datasets

Phase specific datasets for Phase 1, Phase 2 and Phase 3 studies generated for training different models. In order to estimate the performance of our machine learning models, the train-test split method was used^18^. For the final model, a 70:30 train to test split ratio was selected. The train set is used to train the models, whereas the test set is held aside for the final evaluation of the model. This is an effective and fast approach to test our trained models with data they have never seen before.

#### Handling data imbalance

Data imbalance is one of the main challenges of using clinical trials dataset for termination classification. The ratio of positive to negative samples for the overall dataset, which contains studies from all phases, is 15:85. Hence, classification would be biased towards the positive class if the imbalance is not handled. This can result in a falsely perceived positive effect on the model accuracy. Therefore, random under-sampling is applied to the training set. According to the defined positive/negative ratio, a necessary number of data points are deleted from the positive class subset. We use a 1:1 ratio for random under-sampling between the negative and positive class. Random under sampling was applied only on training samples after the train test split. Hence, the test set remained imbalanced to preserve a realistic test distribution.

#### Top feature selection

The feature set size increased significantly due to the addition of eligibility criteria features. In order to achieve the best performance without generating unnecessary noise in the data, feature selection was applied. An ablation study was done to understand the effects of adding more features to the model performance. The number of features vs model error plotted with a purpose to find an elbow point. The elbow point is where the decrease angle of the error line dropped significantly, so that we know adding more features does not have a significant effect on the performance. Once the optimal number of features for training is determined with this method, we selected features according to the k (the number of features needed) highest scores^19^. We used Analysis of Variance (ANOVA) F score as the scoring function^20^.

#### Machine learning model selection

Logistic regression, random forest classifier and extreme gradient boosting classifier (xgBoost) are trained and evaluated. The logistic regression classifier is a simpler algorithm compared to the tree-based ensemble models, such as random forest and extreme gradient boosting^21,22^. Though feature selection is applied, the final datasets are still large sparse datasets. This ruled out many machine learning architectures.

#### Model evaluation

Particularly in imbalanced datasets, splitting the dataset into train and test sets drastically decreases the number of samples used for learning. Hence, fivefold cross validation is used for the model evaluation to achieve unbiased metric scores. The dataset split into 5 smaller sets and the model trained 5 times. The performance of the model reported as the average of 5 experiments, and each time a different chunk is used as the test dataset. This provided reliable metric scores to evaluate different models.

#### Model hyperparameter tuning

Tree based models require careful hyperparameter tuning; however, it is computationally expensive to test every combination of parameters to achieve the best results. Therefore, a strategy is made to find the best possible parameters for the models in hand. In order to prevent overfitting, the initial method is to control the model complexity. Maximum depth of each tree and minimum sum of instance weight needed in each child are the two parameters optimised to control model complexity. Increasing these parameters increases the complexity as well as the risk of overfitting. Furthermore, the second method is to add randomness to make training robust to noise^23^. Subsampling of training instances and subsampling ratio of columns during construction of each tree are optimised. Optimal parameters were chosen after several iterations following this strategy.

#### Model interpretations using Shapley Additive exPlanations

SHAP (SHapley Additive exPlanations) is a framework based on Shapley values, a game theory approach^24^. This method is used to get visual outputs to explain model predictions^25^. SHAP locally explains the feature contributions on individual predictions by connecting optimal credit allocation to local explanations using Shapley values. A base value and an output value are calculated for each plot. Base value is the average model output based on the training data and output value is the overall addition of the Shapley values for each feature for that instance. This allows us to explain the influence of features to the prediction.

## Results and discussion

### Eligibility criteria search features results

McNemar’s Test is a statistical test used for paired nominal data, and can be used to compare predictive accuracy of two machine learning models on the same dataset^26^. In this research it used to test the hypothesis that adding the new set of features will change the model performance with statistical significance. Model 1 trained on only study characteristics features, whereas the feature set of Model 2 also includes the eligibility criteria search features and disease categorisation features. To apply this test, xgBoost models were used and both models trained on the dataset including studies of all phases.

McNemar’s test is based on the 2 × 2 contingency table displayed in Table 2. Our null hypothesis is that none of the two models will perform better than the other. Defined significance level (alpha) for this test is 0.01.

**Table 2:** Contingency table to calculate the chi-squared test statistic for McNemar's test. Model 1 trained on only study characteristics features, whereas the feature set of Model 2 also includes the eligibility criteria search features and disease categorisation features.

	Model 2 correct	Model 2 wrong
Model 1 correct	(a) 20,700	(b) 1642
Model 1 Wrong	(c) 2261	(d) 9178

$$Chi-squared ({X}^{2})=\frac{{(b-c)}^{2}}{(b+c)}$$

Conducting the McNemar test, we get a chi-squared (${X}^{2}$) value of 97.85 and a corresponding p-value of 4.504e-23. The p value calculated is lower than the defined significance level 0.01, hence the null hypothesis stating that the performance of the two models is equal is rejected. Thus, the alternative hypothesis that the performances of the two models are not equal is accepted with a level of confidence p <  < 0.01.

### Machine learning classification results

Table 3 shows the comparison of predictive performances of three different machine learning classifiers. The evaluation metric scores are calculated using fivefold cross validation and xgBoost selected as the best performing classifier.

**Table 3:** Evaluation metric scores for different classifiers; Logistic Regression, Random Forest Classifier, xgBoost Classifier trained in all study phases dataset. Evaluation metrics include ROC AUC (Receiving operating characteristic Area Under the Curve), balanced accuracy, F1 score for class 0 and class specific accuracies (class 0/ class 1).

Classifier/Score	ROC-AUC	Balanced Accuracy	F1 score	Class Accuracies (class 0/class1)
Logistic Regression Classifier	71.60%	66.32%	36.71%	66.78%/65.04%
Random Forest Classifier	75.90%	69.66%	40.40%	71.30%/68.04%
Extreme Gradient Boosting Classifier (xgBoost)	78.32%	70.60%	42.50%	68.69%/72.50%

### Training on phase specific datasets results

**Figure 4:**
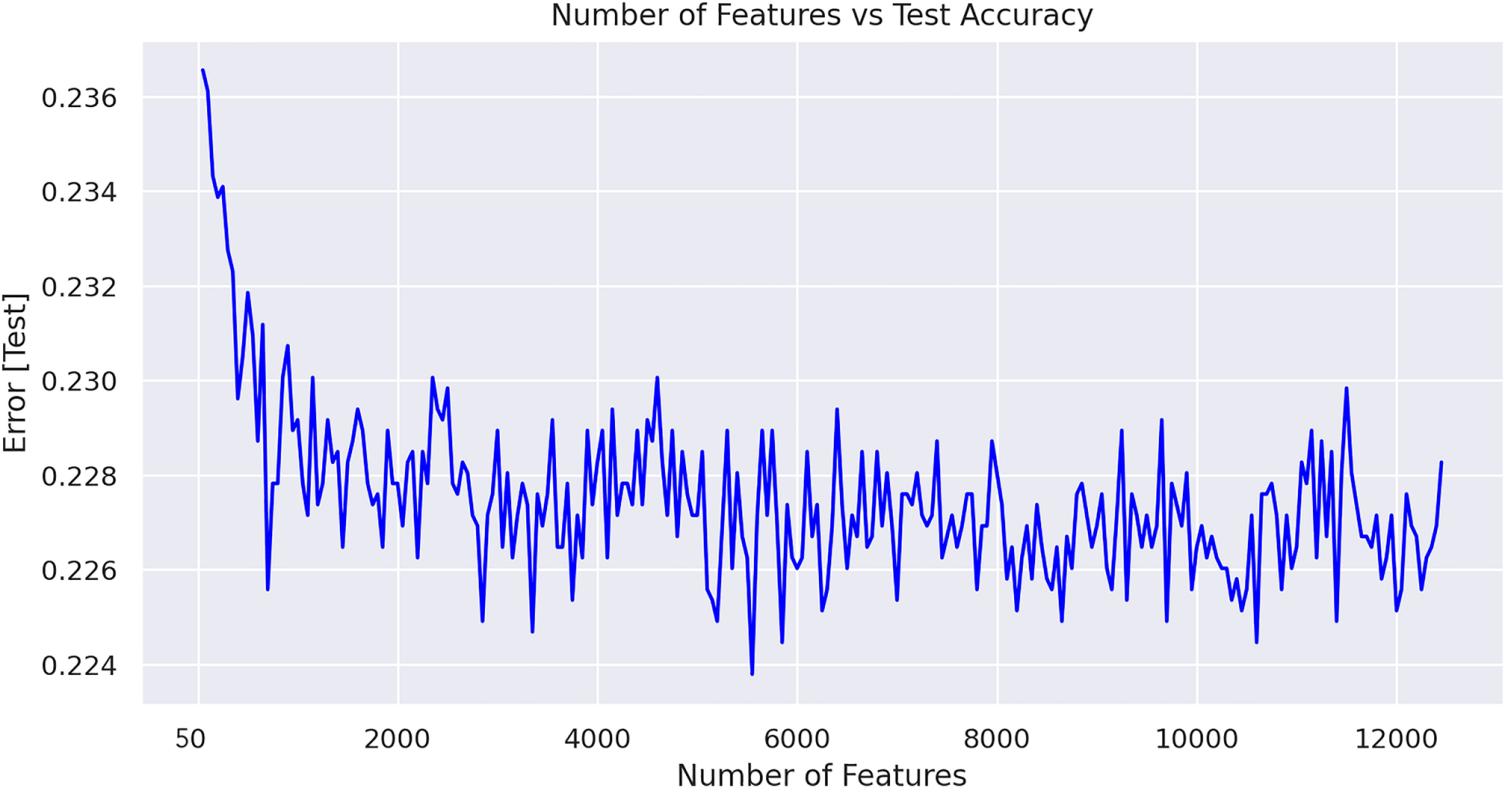
Number of features used for model training vs test error for xgBoost model trained on Phase 2 studies dataset. This plot is used for defining the feature set size by using the elbow point where the decrease angle of the error line dropped significantly, so that we know adding more features does not have a significant effect on the performance.

Phase specific datasets generated, and models trained and evaluated. The feature set is large due to the addition of sparse eligibility search criteria features. Therefore, features selection applied for each phase specific dataset. Figure 4 shows an example plot of number of features vs model error on test set using Phase 2 studies dataset. The purpose of these type of plots is to find an elbow point. Analysing the plot in Fig. 4, we can choose the elbow point as 2,000 features. The same ablation study was conducted for all phase specific datasets.

Table 4 displays evaluation metric scores for xgBoost models trained on the phase specific datasets. We had applied random under sampling on the training set after the train/test splits. Therefore, the test set is imbalanced, and it is significant to evaluate the models using metrics that are independent of class distribution. Model 1 and Model 4 performed similarly and have the highest balanced accuracy scores. On the other hand, Model 2 has the highest F1 score for class 0. In this predictive model, one class is more important than the other since we are trying to predict termination (class 0). Hence, a higher F1 score for class 0 indicates that Model 2 performs better than the others regarding successfully prediction termination probability.

**Table 4:** Clinical trial early termination classification results trained using phase specific (Phase 1, Phase 2, Phase 3 and all phases) subsets. Evaluation metrics include ROC AUC (Receiving operating characteristic Area Under the Curve), balanced accuracy, F1 score for class 0 and class specific accuracies (class 0/ class 1).

	ROC-AUC	Balanced Accuracy	F1 score—Class 0	Class Accuracies (class 0/class1)
Model 1—trained on Phase 1	76.10%	69.61%	39.67%	70.57%/69.90%
Model 2—trained on Phase 2	72.02%	66.23%	48.69%	62.60%/68.88%
Model 3—trained on Phase 3	72.23%	66.32%	38.88%	66.06%/66.69%
Model 4—trained on All phases	79.78%	70.22%	41.80%	70.70%/69.70%

### Comparison with state-of-the-art models

Table 5 demonstrates a comparison of the state-of-the-art models in this domain to ours. All the models used in this comparison are trained using data that includes studies from any phase. Similar to the comparison in the previous section, the most important aspect of model evaluation is the consideration of the data imbalance in the test sets. The model generated by Follett et al., has a significant gap between the F1 score for failed class and overall accuracy resulting in a poor predictive performance as seen in Table 5^9^. Comparing the model generated by Elkin et al. and ours in Table 5, the balanced accuracy and overall accuracies are quite similar, but our model performed higher in ROC-AUC and F1 Score for class 0^10^. This indicates that our model is less biased, considering the data imbalance in this dataset, and performs better in predicting early trial termination.

**Table 5:** Evaluation metric scores for state-of-the-art trial termination prediction models vs ours. Follett et al. used a random forest model trained on study characteristics features and features generated by text mining of study descriptions^9^. Elkin et al. used an xgBoost model trained on study characteristics features and document embedding vector features^10^. In this research we used an xgBoost model trained on study characteristics and eligibility criteria search features generated via CHIA dataset^11^. Evaluation metrics include ROC AUC (Receiving operating characteristic Area Under the Curve), balanced accuracy, F1 score for class 0 and overall accuracy. The dashed table cells represent that information is not provided by the related paper.

	ROC-AUC	Balanced Accuracy	F1 Score	Accuracy
Random Forest Classifier trained on all phases in this study by Follett et al	–	–	3%	93%
Extreme Gradient Boosting Classifier (xgBoost) trained on all phases in this study by Elkin et al	63.92%	67.20%	31.21%	73.01%
Extreme Gradient Boosting Classifier (xgBoost) trained in this study	79.78%	70.22%	41.80%	70.30%

### Interpreting predictions with SHAP

The last stage in Fig. 1, “Interpret Predictions”, is an iterative protocol edit process validated by the final machine learning model selected. Any new clinical study protocol in to our pipeline, will be the input of the xgBoost model pretrained on regarding study phase. If the predicted probability by the model is lower than the defined threshold, interpretation phase will be activated. Using SHAP library, the features contributed to this specific termination probability prediction will be outputted for the clinicians to assess. This will flag the features to reconsider while editing the study protocol into its optimal final state. This process is significantly helpful as eligibility criteria search features are included in these feature contributions, alongside other study characteristics features. For example, if an inclusion or exclusion criteria flagged as a high contributer to failure prediction, this might suggest possible recruitment failure.

For the purpose of demonstrating this workflow, xgBoost model trained on Phase 2 dataset selected as the final model to be used for further investigation using SHAP library. The Phase 2 dataset consists of 8,880 data points and the top 2000 features selected as explained in the feature selection section. Three study indices that the trained model had never seen before were selected randomly from our test set to visualise the prediction interpretations using SHAP values. We limited the number of contributors visualised in the plots to the top 8 contributors in order to visualise effectively. It is also possible to view each feature contribution in detail for every new prediction.

**Figure 5:**
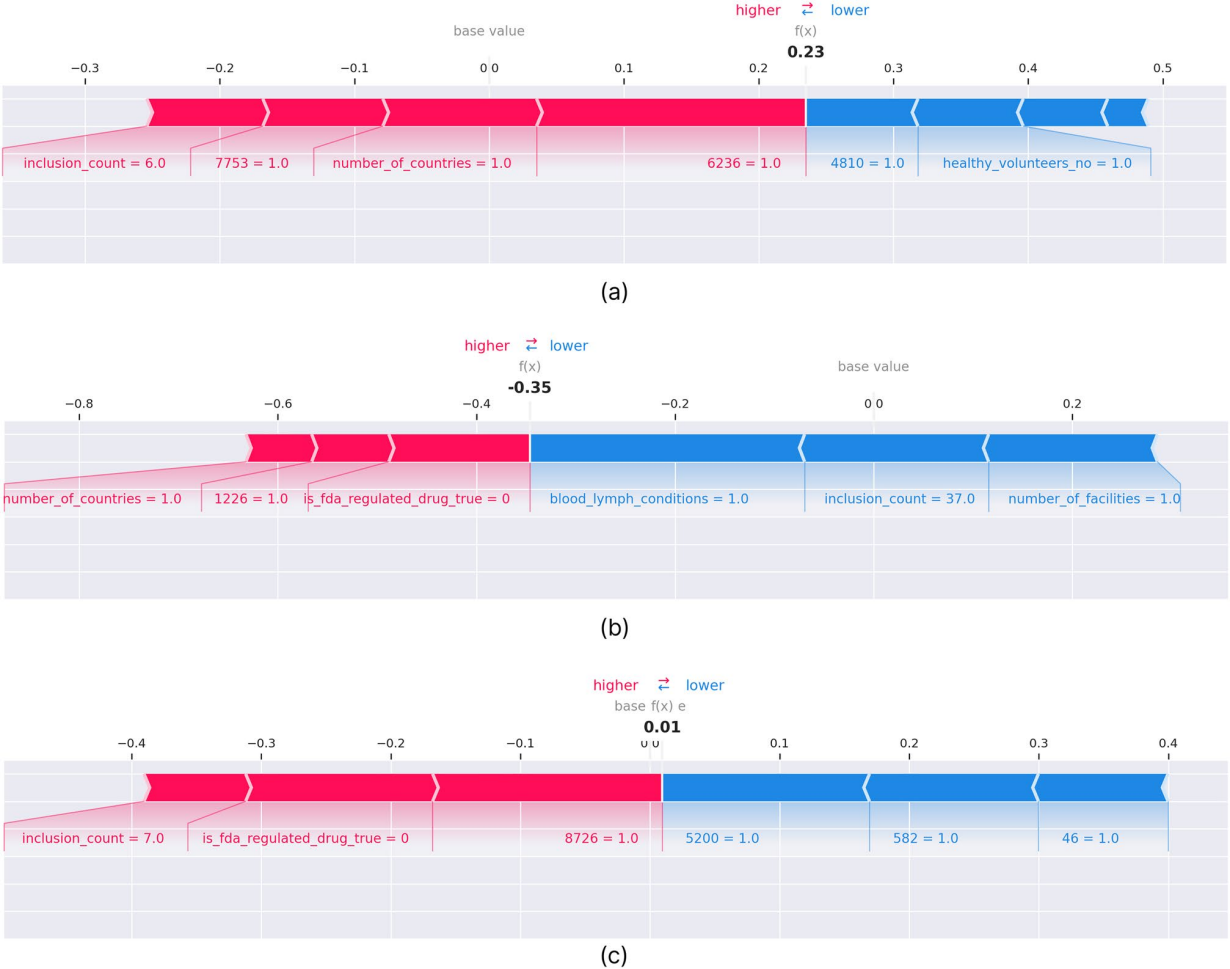
SHAP force plots for 3 test instances to show feature contributions for the model prediction. (**a**) Prediction and actual class are positive; (**b**) Prediction and actual class are negative; (**c**) Prediction and actual class are not the same.

In the SHAP plots for 3 different test instances, pink coloured bars indicate features contributed positively, whereas the blue coloured bars indicate features contributed negatively to the prediction. The overall value is calculated by adding up negative and positive contributions for every feature and then compared to the base value calculated for this model. The base value for this model is -0.00088. The base value is shown in grey text on the scale in, and the calculated value is shown in bold black on top of the scale for each plot in Fig. 5. Figure 5a,b show successful predictions for test cases that belong to Class 0 and Class 1 respectively. Figure 5c shows an unsuccessful prediction where the actual class of the test item and the predicted class are not the same.

As Fig. 5a illustrates, the number of countries, inclusion criteria count value and eligibility criteria features 6236 (exclusion—Condition/inflammation) and 7753 (exclusion—Condition/dry eye) contributed positively, whereas eligibility criteria feature 4810 (exclusion—Temporal/active) and non-acceptance of healthy volunteers contributed negatively. The overall value calculated is 0.23, which is larger than the base value of the trained model, so the outcome prediction is Class 1, which is the accurate classification for this instance. Similarly, Fig. 5b shows an accurate prediction for Class 0, where the calculated contributions add up to -0.71, hence the predicted class is Class 0, the same as the actual class. This example illustrates how this interpretable prediction can be used in a real-world clinical study scenario. The flagged features by the local interpretations will lead to further assessment by the clinician to suggest further edits to the study protocol. This iterative process will be continued until the model validates the protocol and predicts success with low uncertainty.

Figure 5c illustrates an example where the model made a bad prediction. The overall calculated value is 0.01, so the class of the instance is predicted as Class 1, whereas the true class was Class 0. The calculated value and base value are quite similar, but if we take a deeper look at this example, some of the eligibility criteria features that are among the largest contributions for the prediction are slightly illogical. 8726 (exclusion—Condition/addiction) contributed positively and 5200 (exclusion—Condition/inability) contributed negatively, which are intuitive explanations for the prediction, whereas 582 (Inclusion—Mood/confirmed) and 46 (inclusion—Person/age) are not as intuitive, as it is harder to understand the implications, they have for the study protocol. This illustrates that although for some examples SHAP interpretability is an intuitive approach to make suggestions on clinical trial design, there is still room for improvement, especially on eligibility criteria feature engineering.

## Conclusion

In this study, we extracted 420,268 clinical trials from the AACT database and used feature engineering methods on numerical, categorical and free-text columns, and used machine learning to predict early trial termination using these features. We proposed adding eligibility criteria search features generated from free text columns and disease categorization features to the study characteristics features and showed that this approach is statistically significant to increase the early clinical trial termination prediction performance. We achieved 80% ROC-AUC, 70% balanced accuracy and 42% as the F1 score on the xgBoost model trained using all phases dataset. Finally, we used SHAP explanations to transform our black-box machine learning models into insightful suggestions towards the clinical study protocols. Eligibility criteria and design features are flagged as contributions towards success or failure during the interpretation process. These flagged features might help prepare for potential recruitment issues, as well as suggesting direct changes to the study design if possible. Hence, this pipeline provides an optimised machine learning based trial design process.

### Limitations and future work

We used an unstructured eligibility criteria field to generate eligibility criteria search features with the help of the CHIA annotated criteria dataset for 1,000 Phase 4 studies. It is possible that this dataset does not cover some of the main eligibility criteria categories and entities for earlier phase studies. Furthermore, some of the eligibility criteria search pairs generated from the CHIA dataset are not as intuitive as others. There is room for development regarding the generation of search pairs. Once these search features are extended for all phase studies and a more complex processing applied to get the most meaningful search features, it is expected that the predictive power of the trained models will increase, and more intuitive suggestions can be made for the study protocol by interpreting these models.

## Supplementary Information

Supplementary Information.

## Data Availability

As mentioned in the Data Source section, clinicaltrials.gov is a public dataset and AACT is the public database version of clinicaltrials.gov. Similarly, CHIA dataset used in this project is a public dataset and referenced in the corresponding section of the paper.
