# Supplementary material for: Improving clinical trial design using interpretable machine learning based prediction of early trial termination
Source: Sci Rep. 2023 Jan 4;13:121. doi: 10.1038/s41598-023-27416-7 (PMC9813129; doi:10.1038/s41598-023-27416-7)
Supplement: Supplementary file 1 — Supplementary Information. [file 41598_2023_27416_MOESM1_ESM.docx]

**Improving Clinical Trial Design Using Interpretable Machine Learning Based Prediction of Early Trial Termination***Ece Kavalci

[ece@lindushealth.com](mailto:ece@lindushealth.com)

Lindus Health, London, UK

Anthony Hartshorn

[anthony.hartshorn@gmail.com](mailto:anthony.hartshorn@gmail.com)

Lindus Health, London, UK

**Supplementary Material**

**Numeric features in the final feature set**

| **Feature Name** | **Range** |
| --- | --- |
| Number of arms | 1 - 44 |
| Number of sites | 1 - 1746 |
| Number of countries | 1 - 59 |
| Minimum age accepted | 1 - 80 |
| Number of primary outcomes to measure | 1 - 217 |
| Number of secondary outcomes to measure | 1 - 275 |
| Number of Inclusion Criteria | 1 - 109 |
| Number of Exclusion Criteria | 1 - 122 |
| Number of all Eligibility Criteria | 1 - 126 |

Table 1 Numerical feature names and range of values in the final dataset

**Categorical features in the final feature set**

| **Feature Name** | **Sub-categories** |
| --- | --- |
| Responsible Party Type | Sponsor / Principal Investigator/ Sponsor-Investigator |
| Accepted Gender | All / Female / Male |
| Accepts Healthy Volunteers | Binary |
| Allocation | Randomised / Non-randomised |
| Intervention Model | Parallel Assignment / Single Group Assignment / Crossover Assignment / Sequential Assignment / Factorial Assignment |
| Primary Purpose | Treatment / Prevention / Basic Science / Other / Supportive Care / Diagnostic / Health Services Research / Screening / Device Feasibility / Educational - Counselling - Training |
| Masking | None (Open Label) / Single / Double / Quadruple / Triple |
| Intervention Type | Behavioural / Biological / Combination product / Device / Diagnostic test / Dietary supplement / Drug / Genetic / Other / Procedure / Radiation |
| Is FDA regulated device | Binary |
| Is FDA regulated drug | Binary |
| Subject Masked | Binary |
| Caregiver Masked | Binary |
| Investigator Masked | Binary |
| Outcomes Assessor Masked | Binary |
| Condition Category | see Figure 2 in Manuscript |

Table 2 Categorical feature names and their sub-categories in the final dataset
